# Communicative roles in human-anchored and AI-presented news commentary short videos: a multimodal intertextuality analysis

**DOI:** 10.3389/fpsyg.2026.1890944

**Published:** 2026-09-07

**Authors:** Xuanli Li, Xunian Wang

**Affiliations:** 1 Postgraduate School, Xi'an International Studies University, Xi'an, China; 2 Center for Linguistics and Applied Linguistics, Guangdong University of Foreign Studies, Guangzhou, China

**Keywords:** communicative roles, human-AI collaboration, intertextuality, multimodality, news commentary short video

## Abstract

**Introduction:**

Despite the growing integration of artificial intelligence (AI) into news production, little is known about the intertextual differences between human and AI anchors, the communicative roles constructed through such differences, and their multimodal realization in short-video news commentaries.

**Methods:**

This study approaches multimodal intertextuality from a social semiotic perspective and employs a quantitative multimodal content analysis to compare human-anchored and AI-presented news commentary short videos.

**Results:**

The findings reveal that though both programs rely predominantly on verbal intertextuality, human-anchored videos employ a more diversified multimodal intertextual repertoire, particularly through commenting and evaluating and genre intertextuality, whereas AI-presented commentaries depend heavily on direct quotation and make comparatively limited use of other intertextual techniques. These contrasting intertextual configurations construct distinct communicative roles.

**Discussion:**

Human-anchored news more frequently assumes the role of an interpretive mediator, recontextualizing information through evaluative, explanatory, and genre-based intertextual practices. By contrast, AI-presented commentaries primarily functions as an institutional information relay that emphasizes fidelity to source materials and authoritative information transmission. Moreover, the multimodal analysis indicates that AI-presented commentaries exhibit a narrower range of verbal-visual intertextual interaction than their human-anchored counterparts, suggesting that technological sophistication in presentation does not necessarily translate into greater intertextual diversity. These findings point to an emerging division of communicative labor in contemporary journalism, highlighting the importance of role differentiation and collaboration in the development of human-AI news production.

## 1 Introduction

Journalistic roles have traditionally been examined as professional norms and institutional expectations. More recently, however, scholars have argued that roles should also be understood as performances manifested through news discourse itself (Mellado, 2015; Mellado and Van Dalen, 2014). From this perspective, communicative roles are not merely predefined professional identities but are actively constructed through textual and multimodal practices. Simultaneously, the increasing integration of AI technologies into news production has prompted growing scholarly interest in AI as a communicative actor rather than a purely technical tool (Lewis et al., 2019; Guzman and Lewis, 2020).

News commentary, the practice whereby media outlets reconstruct and critique newsworthy events, has expanded in the digital era to include a wide range of voices, from mainstream journalists to social media users, who post briefs foregrounding value judgments (Kim, 2023, p. 869). This ecosystem now includes highly popular short videos, a format where AI-powered virtual anchors have emerged alongside human anchors to enhance production efficiency. The rise of AI news anchors, tracing to 2000 and marked by the 2018 launch of the first synthetic anchor (Xue et al., 2022, p. 1), has been accelerated by advances in AI-generated content (Liu et al., 2025). While proponents cite their ability to process data and deliver accurate reports (Li, 2025, p. 6), critics point to a delivery that “deviates from a humanoid approach” and “is very flat,” and “has no rhythm, pace, or emphasis” (Wiederhold, 2019, p. 675). Rather than viewing AI as a replacement for human journalists, recent scholarship increasingly conceptualizes journalism as a site of human-AI collaboration, raising questions about how communicative responsibilities may be distributed between human and AI anchors.

The communicative role of news commentary has attracted increasing scholarly attention. While striving for objectivity, news outlets inevitably convey ideology through framing and emphasis (Charteris-Black, 2004, p. 115; Fowler, 1991, p. 4), as news language is neither “neutral” nor “value-free” (Krennmayr, 2014, p. 530). This inherent subjectivity facilitates public-opinion guidance, for which intertextuality, the embedding of prior texts through quotation, allusion, and paraphrase, is a central mechanism. Research shows intertextuality can “create conceptual metaphors” to enrich meaning (Velykoroda and Moroz, 2021, p. 60), “create an emotional or expressive effect” (Hart, 2017), or serve to “manipulate the recipient” and “misinform readership” (Morgan, 2019; Saraireh and Saraireh, 2020). The role of intertextuality is further complicated by AI, whose outputs constitute a “mosaic of quotations” reconfigured from training data (Tang, 2025, p. 9), making AI news an inherent intertextual assemblage. As AI technologies become increasingly integrated into news production and presentation, understanding how AI-presented and human-anchored programs organize and apply intertextual techniques has become an important issue for contemporary media and communication research. As questions concerning communicative role construction and human-AI collaboration become increasingly important, examining them within specific media systems is essential. In China’s contemporary media landscape, the government prioritizes opinion guidance on social media to counter misinformation and maintain stability (Li, 2024; Xinhua News Agency, 2015; Wells, 2023). In response, mainstream media have expanded their social media presence (Ai et al., 2018). However, the strategic variations between commentaries and their source texts, are poorly understood (Bazerman, 2004, p. 83). Nevertheless, because intertextual practices are often shaped by genre-specific conventions and communicative purposes, the applicability of existing frameworks to emerging forms of AI-presented news commentary cannot be assumed. Against this backdrop, several critical gaps persist: a scarcity of quantitative comparisons of intertextuality between human- and AI-presented news commentaries, limited understanding of how different intertextual configurations contribute to communicative role construction, a general lack of multimodal intertextual analyses of short-video news commentaries, and insufficient attention to what these differences reveal about emerging forms of human-AI communicative collaboration.

This study addresses these gaps by conducting a comparative intertextual analysis of two short video commentary programs from 央视屏(*Yang Shi Ping*): 主播说联播(*Zhubo Shuo Lianbo*, hereafter ZSL, human-anchored) and “冠”察财经(*“Guan” Cha Caijing*, hereafter GCC, AI-presented). Because both programs are produced by the same media institution yet differ in presenter type, they provide a particularly suitable basis for analyzing the communicative meaning constructed in human- and AI-presented programs. Adapting a social semiotic perspective and Bazerman’s (2004) framework, the study systematically compares the frequency, distribution, and multimodal interactions of intertextual techniques across the two programs. The study investigates how multimodal intertextual configurations shape communicative role performance and what these interactive patterns reveal about the emerging division of communicative labor between human and AI presenters.

RQ1: How do AI-presented and human-anchored financial news commentaries differ in their use of multimodal intertextual resources?

RQ2: What communicative roles are constructed through these different multimodal intertextual interactions?

RQ3: What do these differences suggest about the emerging division of communicative labor between human and AI news presenters?

## 2 Theoretical framework

This study adapts Feng’s (2023) social semiotic-based multimodal discourse analysis theoretical framework and integrates it with a intertextuality analysis framework, thus forming a multimodal intertextuality analysis framework. Social semiotics, which examines how society is represented through symbolic systems, provides an effective holistic framework for the comprehensive analysis of meaning construction in multimodal communication (van Leeuwen, 2005; Kress and van Leeuwen, 2006; Kress, 2010). From the perspective of social semiotics, the symbol producers create different meaning patterns through a series of symbolic resources shaped by social culture. Therefore, this practice is also influenced by the social and historical background (Kress, 2010). The analysis of multimodal symbolic resources can help reveal the underlying social communication relationships.

Intertextuality theory, which reveals dialog and connections between texts through quotation, allusion, and structural borrowing (Kazmierczak, 2019, p. 364), provides a robust framework for examining how communicative roles are constructed through the transformation and recontextualization of prior texts. Rooted in Bakhtin (1986, p. 92) dialogism, the concept has been developed by scholars who define it as the transformation of historical texts into new contexts (Bhatia, 2010, p. 35) and famously characterize every text as a “mosaic of quotations” (Kristeva, 1969, p. 146). This perspective underscores that all language responds to and transforms earlier meanings, and no discourse exists in isolation (Kristeva, 1986, p. 37; Vološinov, 1986, p. 86). Consequently, news commentaries can be understood not merely as reports on events but as discursive sites where existing meanings are selectively recontextualized and reinterpreted. While various classifications of intertextuality exist (e.g., Fairclough, 1992; Kristeva, 1969), this study adopts Bazerman’s (2004, p. 86–89) framework for its analytical clarity. Among existing approaches, Bazerman’s (2004) typology is particularly useful because it captures both explicit and implicit forms of intertextuality, which is suitable for news commentary because the genre is fundamentally built upon the transformation of prior news materials. Through different intertextual techniques, anchors do not simply reproduce source information but reorganize, evaluate, and contextualize it in ways that contribute to the construction of distinct communicative roles. In the new media environment, such transformations frequently extend beyond linguistic borrowing to include the incorporation of visual and discursive resources from multiple media contexts. Since news commentary videos are inherently multimodal, the linguistic intertextuality cannot explain whether or to what extent the non-textual modality is associated with the textual modality and constitutes an intertextual whole together.

Moreover, the framework has been widely applied across diverse communicative settings, ranging from business communication (Bremner, 2008) to multimodal discourse such as YouTube tutorials (Bhatia, 2018) and commercial videos (Xing and Feng, 2023). These studies provide the foundation for this article to adapt Bazerman (2004) intertextual classification to a multimodal context. Thus, within the above social semiotic based framework, this study regards the linguistic modality and visual modality as a unified set of symbolic resources used for constructing meaning. This multimodal extension is grounded in the principle that intertextuality operates across all semiotic systems (Forstall and Scheirer, 2019, p. 113). Such an approach is particularly relevant for contemporary short videos, where communicative roles are realized through the interaction of language, visual imagery and graphic design. We define “multimodal” from a semiotic standpoint, drawing on Kress (2010, p. 87) concept of a mode as a socially shaped resource for meaning-making. This definition itself highlights the intertextual nature of modalities. Following van Leeuwen (2005, p. 3), we treat semiotic resources as the specific, producible devices for communication. Although Bazerman’s (2004) typology of intertextuality was originally developed for linguistic mode, the techniques themselves are defined according to the communicative meaning through which a semiotic resource relates to other resources, rather than the linguistic forms by which those relations are expressed. Following Bazerman (2004), the six multimodal intertextual techniques are operationalized as follows:

1. Direct Quotation (DQ): The unmodified inclusion of multimodal elements from other news.
2. Indirect Quotation (IQ): Using symbolic resources that are thematically related to a news event but do not replicate a specific source.
3. Mentioning (MEN): Referencing a person, document, or statement through symbols without detailing its content, presupposing audience familiarity.
4. Commenting and Evaluating (CE): Presenting symbolic resources that frame the commentary with a specific attitude or judgment.
5. Phrasing or Terminology (PT): Incorporating symbolic resources of other discursive communities atypical of standard news communication, thereby temporarily shifting the video’s contextual register.
6. Genre Intertextuality (GEN): Employing presentational forms and structures constitutive of a particular communication rule. This is a more macroscopic technique, echoing other texts in genre and style.

Together, these techniques enable the analysis of both micro-level symbolic transformations and broader patterns of multimodal intertextuality. Accordingly, the analytical procedure consisted of the identification of multimodal intertextual techniques, the exploration of interaction pattern and the interpretation of the construction of communicative meaning and roles is detailed in the following section.

## 3 Methodology

### 3.1 Dataset collection

The term AI-presented news in this study refers to news commentary delivered by a virtual AI anchor. Importantly, GCC adopts a human-AI collaborative workflow in which AI technologies assist in content generation and presentation, while editorial personnel remain responsible for content review, verification, and publication decisions. Consequently, the study examines AI-presented commentaries as a multimodal communicative product emerging from a human-AI collaborative production process. This study employs a two-step quantitative design. Step 1 conducts a comparison of multimodal intertextual features in short videos, focusing on the financial theme to contrast the AI-presented program GCC with the human-anchored program ZSL. Step 2 expands the quantitative analysis of multimodal intertextual techniques using a larger sample from ZSL covering more themes to provide a comprehensive profile of human-anchored commentary. The two-step design was adopted to balance comparability and representativeness. Step 1 establishes a thematically controlled comparison between AI-presented and human-anchored news commentaries, while step 2 broadens the analysis to examine the wider intertextual repertoire of human-anchored commentary across diverse topics to provide insight for human-AI collaboration.

The program selection is strategic, controlling for the authoritative media source variable, as both are produced by China Central Television Group and serve as official commentary products within China’s mainstream media system. Selecting two programs from the same media institution reduces variation attributable to organizational culture, editorial policy, and platform characteristics, thereby facilitating a more focused comparison of intertextual practices. The AI anchor “AI Wang Guan,” in GCC, modeled on financial commentator Wang Guan using deep learning, is an representative AI news anchor (Global Times, 2022). Integrated with global financial data, it produces daily streams, forming a macro-policy interpretation and data analysis model. The GCC short video series serves to interpret financial data and comment on economic situations, aiming to influence audience perception. Its content originates from official Chinese fiscal documents, major socio-economic events, and expert analyses. ZSL features human anchors who comment on news taken directly from the authoritative news program *Xinwen Lianbo* (新闻联播, hereafter XL), which is popular in China due to the status and influence (Hou, 2021). Both programs are paradigmatic examples of mainstream media adapting to new media forms, allowing them to inherit the authority of their source programs while adopting social media characteristics.

For the comparative analysis of intertextuality between AI and human anchors, data were systematically collected from the official “*Yang Shipin* (央视频)” platform.^1^ GCC short videos was selected from videos published between January 1 and June 30, 2023. This period was chosen because it followed the program’s 2023 upgrade and represented the first stable phase of its updated production format. After compiling a sampling frame of all eligible 77 videos and assigning each a unique identification number, simple random sampling without replacement was conducted in R to select 30 videos. This procedure ensured reproducibility and gave every eligible video an equal probability of inclusion. Each video has an average duration of 1 min 13 s, and focuses exclusively on financial news commentary, and was transcribed into 9,978 Chinese characters. To construct a comparison corpus, 22 ZSL videos published during the same period were selected through purposive matching. Only short video news commentaries whose primary focus concerned economic and financial issues, including macroeconomics, finance, industry policy, market regulation, trade, and economic development, were included. The final ZSL corpus (ZSLeco) consisted of 22 videos with a mean length of 1 min 58 s and 11,505 transcribed Chinese characters. Comparability between the two corpora was ensured through alignment in publication period, thematic domain, communicative genre, short video media format, and corpus scale, thereby providing a suitable basis for cross-program comparison.

To extend the analysis of intertextual features across diverse topics in human-anchored commentary in step 2, all ZSL short videos published within the period was analyzed to fulfill a holistic analysis of multimodal intertextuality. The initial ZSL corpus comprised 167 videos. One outlier video (posted May 1, 2023; duration > 9 min; featuring three anchors summarizing their work) was excluded. The final expanded ZSL corpus thus contains 166 videos (mean length = 2 min 17 s, SD = 0.34), transcribed into 86,576 Chinese characters. The corresponding source news reports for all commentaries were retrieved from the open-source XL website.^2^ The platform’s automatic segmentation of each program into semantic blocks facilitated precise intertextual tracing. All materials are publicly accessible and are partially cited in this paper for reasonable and necessary illustrations.

### 3.2 Analysis procedure of multimodal intertextuality

The analysis content of this study enables a systematic examination of how intertextual resources are distributed across linguistic and visual modes and how these resources contribute to different forms of commentary construction in human-anchored and AI-presented news programs. The identification process is conducted according to the adapted Bazerman’s categorization, while the interpretation process is based on the visual grammar proposed by Kress and van Leeuwen (2006). Based on Halliday’s (1994) three major language metafunctions, they proposed that visual images also possess three metafunctions, namely representational meaning, interactive meaning, and compositional meaning. Representational meaning contains narrative representation and conceptual representation, whose distinction lies in the relationship between visual image participants. The former features an unfolding of actions and events, processes of change, while the latter a generalized, stable and timeless essence (Kress and van Leeuwen, 2006). In the news commentary videos, narrative representation can explain the active and passive roles in the process of news commentary. Conceptual representation defines the content of news commentary by explicitly or implicitly depicting the relationships among the visual image participants. Interactive meaning consists of three semantic categories, that is, contact, social distance and attitude (Kress and van Leeuwen, 2006), which can be used to specify the relationship between visual image participants such as the image of the anchors and news commentary video audiences. The information value, salience and framing in compositional meaning integrates the representational meaning and interactive meaning into a meaningful whole (Kress and van Leeuwen, 2006). The communicative meaning reflected by the technical configuration of intertextual symbols in news commentary videos can be explained through compositional meaning.

### 3.3 Coding procedure

For Linguistic mode intertextuality, the coding unit was defined as one subtitle block. Subtitle blocks were retained in their original form as generated across both GCC and ZSL corpora. This criterion was selected because intertextual references frequently operate at lexical, phrasal, and clausal levels, while sentence boundaries in the subtitles were often unclear due to incomplete punctuation and frequent line breaks. Multiple coding was permitted when a unit simultaneously exhibited more than one intertextual feature. Multiple units may be simultaneously coded as one intertextual technique when they form a unifies semantic chunk. To assess the robustness of the findings, frequency comparisons were additionally aggregated at the video level, reducing potential effects of subtitle segmentation on statistical results. The situation of dialog with the source information is conducted through fuzzy matching search based on the key words of the transcribed text content, and the existence and type of intertextuality in the short video news comment content are determined through manual comparison. For information that cannot be retrieved, no encoding is performed. Table 1 specifies the coding manual for each intertextual category. For units that conform to the definition of intertextual techniques, they are labeled as 1 under this type of technique. Units that do not belong to any intertextual technique (IT) are labeled as 0.

**Table 1:** Coding manual of textual mode intertextuality.

IT	Operational definitions	Examples	Source
DQ	Verbatim reproduction of words, phrases, slogans, statements, or expressions originating from an identifiable external source.	2. 拼多多去年 3. 全年研发费用 4. 同比增长15% (*Last year, the R&D expenditure of Pinduoduo increased by 15% year-on-year.*)	GCC
IQ	Reformulation or paraphrasing of information, opinions, or statements originating from an external source without reproducing the exact wording.	14. 今天新闻联播的 15. 头条新闻 16. 就是昨天下午 17. 中共中央政治局进行的 18. 第二次集体学习 (*Today’s top story on the XL was the second collective study session held by the Political Bureau of the CPC Central Committee yesterday afternoon.*)	ZSL
MEN	Explicit references to external persons, institutions, organizations, events, documents, locations, media outlets, or social actors without reproducing their discourse.	37. 有一位务工人员 38. 在接受采访时就说 39. 想把自己的生活水平 40. 提高一点 41. 多挣点钱 (*A migrant worker said in an interview that he wanted to improve his standard of living and earn more money.*)	ZSL
CE	Expression of judgments, attitudes, assessments, or evaluations toward an external discourse, event, actor, or issue.	5. 真的是一个 6. 非常巨大的数字 (…*is truly an extremely large figure.*)	ZSL
PT	Borrowing, adaptation, or recontextualization of recognizable linguistic expressions, rhetorical formulations, idioms, slogans, metaphors, or discourse patterns from prior texts.	6. 我们东北那儿 7. 是老漂亮 8. 老好玩了 (*That part of the northeast was literally beautiful and literally fun.*)	ZSLeco
GEN	Incorporation, imitation, blending, or invocation of conventions associated with another communicative genre.	4. 这个年您是怎么过的呢 5. 是回家团聚了 6. 还是外出游玩了 7. 欢迎大家留言 8. 和我们来说一说啊 (*How did you spend this New Year Festival? Did you go back home to reunite with your family or go on an outing? We welcome everyone to leave a message and tell us about your experiences.*)	ZSLeco

The examples in Table 1 are presented in the form of subtitle units, and the numbers indicate the subtitle position of these units in the video. For each case, a italicized literal English translation is provided below. All coding work is conducted by a researcher of this study. Then, a second trained coder independently coded a randomly selected 20% subsample, comprising six GCC videos and five ZSL financial-themed videos. Intercoder reliability was assessed using Cohen’s kappa for categories with sufficient variation in coding decisions. For GCC, kappa was calculated for DQ, the most frequently occurring category. For ZSLeco, kappa was calculated for MEN, CE, and GEN, which contained adequate numbers of positive instances for reliable estimation. All kappa coefficients exceeded the commonly accepted threshold of 0.80, indicating substantial agreement. For techniques with extremely low prevalence, Cohen’s kappa was not considered informative because of its sensitivity to highly imbalanced distributions. Instead, percentage agreement was examined, with all remaining techniques achieving agreement rates above 95%.

For visual mode, ELAN 6.7 (Lausberg and Sloetjes, 2009) was used to annotate three visual tiers: video, picture, and special effect. A coding unit was defined as one continuous visual element (e.g., from the appearance of a video clip to the disappearance of the clip) that contributed to the commentary and could be classified into one of the six intertextual categories. The identification visual intertextuality was guided by its relationship to the accompanying verbal commentary rather than by visual form alone. A continuous visual resource was counted as a single occurrence regardless of internal edits or camera cuts. Each video was repeatedly reviewed, coded, and the results were exported and merged into a single dataset for subsequent quantitative analysis. Since the intertextual phenomenon of visual modes are easy to be classified based on the verbal content of the commentary video, the coding result was double checked by two researchers, making sure a proper coding result. Any coding disagreements were subsequently reviewed and resolved through discussion between the two coders before finalizing the dataset.

## 4 Results

### 4.1 Intertextual techniques in different news commentaries

To examine how AI-presented and human-anchored news commentaries differ in their use of intertextual resources (RQ1), the frequencies of intertextual techniques were compared across the two programs. Table 2 presents the raw frequencies of each technique across linguistic and visual modes, revealing notable differences in the distribution of intertextual resources. Linguistic mode intertextuality of two programs were further standardized by the number of textual units within each video. Table 3 reports the normalized frequencies per 100 textual units (mean ± SD), allowing a more reliable comparison of the textual intertextual repertoires of GCC and ZSL.

**Table 2:** Frequency of multimodal intertextuality in GCC and ZSLeco.

Program	Symbolic resource	DQ	IQ	MEN	CE	PT	GEN
GCC	Text	474	5	0	2	3	0
	Picture	1	0	0	0	0	0
	Video	0	1	8	1	0	0
	Special effect	118	0	0	1	0	0
ZSLeco	Text	39	23	47	89	13	107
	Picture	1	1	2	0	0	0
	Video	16	19	42	0	0	0
	Special effect	25	2	2	41	37	5

**Table 3:** Standardized frequency of linguistic intertextuality of GCC and ZSLeco.

Program	DQ	IQ	MEN	EC	PT	GEN
GCC	63.97 (10.6)	0.77 (2.19)	0	0.27 (1.06)	0.42 (1.3)	0
ZSLeco	3.27 (1.43)	1.95 (0.72)	4.02 (1.33)	7.57 (2.06)	1.12 (1.14)	9.02 (1.38)

From Table 3, GCC demonstrated a strong concentration of DQ, with DQ occurring at a mean rate of 63.97 per 100 textual units (SD = 10.60), whereas the corresponding rate in ZSL was considerably lower (M = 3.27, SD = 1.43). However, other intertextual techniques were rarely observed in GCC, while MEN and GEN were absent from GCC at the textual level. In contrast, ZSL exhibited a more diversified use of textual ITs. Overall, the normalized frequencies suggest that GCC relied predominantly on DQ as its primary textual intertextual strategy, whereas ZSL employed a broader range of intertextual resources, particularly evaluative and genre-related techniques.

To examine whether the differences observed in normalized frequencies were statistically significant, video-level comparisons were conducted using Wilcoxon rank-sum tests. Rather than treating individual intertextual instances as independent observations, each video was treated as the unit of analysis, with frequencies normalized by the number of textual units. As shown in Table 4, significant differences were identified across all six textual ITs between GCC and ZSLeco.

**Table 4:** Video-level comparison of linguistic intertextuality between GCC and ZSL.

Technique	GCC Median	ZSL Median	W	*p*	r
DQ	62.62	3.42	660	< 0.001	0.85
IQ	0	1.85	101	< 0.001	0.63
MEN	0	3.70	0	< 0.001	0.94
CE	0	7.41	1	< 0.001	0.92
PT	0	1.64	200	0.002	0.42
GEN	0	9.18	0	< 0.001	0.94

GCC demonstrated a significantly greater reliance on DQ than ZSLeco (*p* < 0.001). By contrast, ZSLeco exhibited significantly higher frequencies for all other linguistic ITs as shown in Table 4. Effect sizes ranged from moderate to large (*r* = 0.42–0.94). In sum, the video-level analysis reveals two distinct linguistic intertextual profiles. AI-presented GCC relied predominantly on DQ as its principal means of incorporating authoritative voices, whereas human-anchored ZSLeco employed a more diversified repertoire. These contrasting patterns of intertextuality provide an empirical basis for the subsequent analysis of communicative role construction.

Because linguistic and visual intertextual techniques were coded using different analytical units, different statistical procedures were applied to the two kinds of modalities. The multimodal intertextuality analysis of financial news commentaries in GCC and ZSLeco reveals notable differences in the distribution of intertextual resources across modes. As shown in Table 2, the configuration of visual intertextuality differed substantially. ZSLeco employed a wider range of visual mode intertextual techniques, particularly through MEN, IQ, CE, PT, and GEN. By contrast, GCC made comparatively limited use of picture and video-based intertextuality, with only isolated instances identified in these modes. Its principal visual intertextual strategy was the use of special effects, which were overwhelmingly associated with DQ (118 instances), suggesting a tendency to visually foreground quoted information rather than diversify intertextual resources across modalities. Overall, while both programs were predominantly text-oriented, ZSLeco exhibited a more diversified multimodal intertextual practice that integrated verbal and visual resources, whereas GCC relied heavily on direct quotation in both textual and special-effects modes. These contrasting multimodal patterns suggest different approaches to source-text recontextualization and foreshadow the distinct communicative roles discussed in the following section.

### 4.2 Distribution of intertextual techniques in the expanded ZSL

Table 5 presents the raw frequencies of multimodal intertextuality identified across the 166 ZSL videos. A total of 3,130 linguistic intertextual instances were identified. Genre-based intertextuality (*n* = 677) and CE (*n* = 684) were the two most frequently employed techniques, together accounting for more than half of all textual IT occurrences. MEN (*n* = 312), DQ (*n* = 276), and IQ (*n* = 264) occurred at moderate frequencies, whereas PT was the least frequent category.

**Table 5:** Frequency of multimodal intertextuality in expanded ZSL corpora.

Symbolic resource	DQ	IQ	MEN	CE	PT	GEN
Text	276	264	312	684	144	677
Picture	191	4	5	0	0	0
Video	207	0	24	0	0	0
Special effects	0	0	0	86	0	256

To control for variation in video length, frequencies of linguistic mode were further normalized per 100 textual units. The results revealed a pattern largely consistent with the raw frequency distribution. CE remained the most prevalent intertextual technique, closely followed by genre-based intertextuality. The similarity between the raw and normalized distributions suggests that the predominance of comment and evaluation and genre-based intertextuality cannot be attributed solely to differences in video length. Rather, these two techniques appear to constitute the dominant linguistic intertextual resources employed throughout the expanded ZSL corpus.

### 4.3 Linguistic intertextuality of news commentary short videos

While the quantitative analysis revealed significant differences in the frequency and distribution of intertextual techniques, frequency data alone cannot fully explain how these resources contribute to meaning-making. To further examine the communicative functions of intertextuality, following parts presents representative examples from linguistic and visual modes, illustrating how different intertextual techniques are mobilized to recontextualize source materials and structure news commentary. An analysis of the interactive construction of communicative meaning in news commentary videos through the intertextuality of linguistic and visual elements can help answer research question 2.

(1) 总书记用“三个事关” (*The General Secretary used “three concerns”*)

事关我国生态安全 (*concern of the ecological security in our country*).

事关强国建设 (*concern of building a strong country*).

事关中华民族永续发展 (*concern of sustainable development of the Chinese nation*).

来深刻概括防沙治沙的重要意义 (*to deeply summarize the significance of prevention and control of desertification*).

(Source: ZSL: *总书记用“三个事关,”深刻概括这项工程的意义*(*General Secretary Used “Three Concerns,” to Deeply Summarize the Significance of This Project*), Date: June 6, 2023)

(2) 4月3日 (On *3 April*)

中国人民银行发布 (*The People’s Bank of China issued*).

2023年 (*The 2023*).

第一季度针对银行家 (*questionnaire survey report for bankers*).

企业家和城镇储户的 (*entrepreneurs and urban depositors*).

问卷调查报告 (*in the first quarter*).

数据显示… (*The data displays that …*).

(Source: GCC: *央行:一季度经营景气指数环比上升2.6%(The central bank: The business sentiment index rose by 2.6% quarter-on-quarter in the first quarter)*, Date: April 4, 2023)

In Example (1), which corresponds to an XL report on a desert governance symposium, ZSL directly quotes Chinese official’s original statements (lines 2–4) while simultaneously introducing the anchor’s own summary frame “three concerns” (lines 1, 5). This synthetic phrase, absent from the original XL report, demonstrates the program’s strategic integration of direct and indirect intertextual resources. In contrast, the feature of DQ, as shown in line 7 of example (2), is clearly evident and occurs frequently in the GCC samples. And lines 1–6 are indirect quotations that introduce the data source. These approaches excessively prioritize informational authority without substantially differentiating from traditional mainstream media narratives.

MEN in ZSL operates on presumed shared knowledge. This technique is deployed when anchors presuppose sufficient audience familiarity with a source, such that mere reference to a person, document, or statement evokes contextual resonance. This shared contextual ground effectively prompts audiences to align with the group identity constructed by the anchor.

(3) 这一年 (*This year*)

面对疫情 (*facing the epidemic*).

经过艰苦卓绝的努力 (*after painstaking efforts*).

我们战胜了前所未有的困难和挑战 (*we have defeated unprecedented difficulties and challenges*).

现在曙光就在前头 (*now the dawn is in front of us*).

大家再加把劲 (*everybody try harder*).

坚持就是胜利 (*perseverance means victory*).

这一年有太多梦想开花结果 (*this year so many dreams have come to fruition*).

冬奥、天宫、第三艘航母 (*Olympic Winter Games, Heavenly Palace, the third aircraft carrier*).

大飞机等等 (*giant plane and so on*).

这离不开每一个你的 (*they are inseparable from each of you*).

辛勤付出和汗水 (*hard work and sweat*).

所以新年到来的时候 (*so when the New Year comes*).

我们可以给自己点个赞 (*we can give ourselves a Like*).

(Source: ZSL: *字字温暖!在新年贺词里体悟不凡的中国* (*Every word is warm! Experience the extraordinary China in the New Year message*), Date: January 2, 2023)

Example (3) demonstrates the strategic application of mentioning. In lines 9–10, the anchor introduces four noun phrases referencing China’s recent achievements, presupposing audience familiarity with these concepts. This technique creates what Bazerman (2004) identifies as interpretive space for position-taking without explicit argumentation. The deliberate lack of specificity serves to cultivate in-group alignment among knowledgeable viewers.

Commenting and evaluating constitute the most frequent textual intertextual technique in ZSL, reflecting the program’s inherent need to assess events through intertextual engagement. In practice, evaluation in ZSL typically combines referenced information with explicit stance-taking, as anchors rarely comment in an informational vacuum. In GCC, by contrast, CE is remarkably scarce. This limitation is particularly paradoxical given the program’s positioning at the forefront of AI technology and impedes audience comprehension of the specific implications underlying financial data.

(4) 今年前四个月外贸数据剬布了 (*The first four months of this year’s foreign trade data have been released*)

同比增长5.8% (*year-on-year growth rate was 5.8%*).

相比于一季度4.8%的增速 (*compared to the first quarter’s 4.8% growth rate*).

又取得了不小的进步 (*no small progress has been made*).

成绩不错 (*score good marks*).

(Source: ZSL: *5.8%背后,哪些“科目”越来越好?* (*Behind 5.8%, which “subjects” are getting better?*), Date: May 9, 2023)

(5) 先来卖个关子 (*I will firstly create a suspense*)

小伙伴们知道 (*do you little friends know*).

今天是什么日子吗 (*what day is it today*).

今天是二十四节气当中的 (*Today is one of the 24 solar terms*).

谷雨 (*Grain Rain*).

也是第十四个 (*also the 14th*).

联合国中文日 (*UN Chinese Language Day*).

(Source: ZSL: *当谷雨与联合国中文日相遇, 遇到的不止是美*(*When Grain Rain meets the UN Chinese Language Day, it is not only beauty that is encountered*), Date: April 20, 2023)

In example (4), lines 1–2 directly quote official statistics from XL without modification, while line 3 introduces an additional statistic through indirect quotation. This syntactically adjusted contrast strategically frames the subsequent positive evaluation in lines 4–5. The quantitative prevalence of this pattern reinforces evaluation as the core mechanism of communication in ZSL.

Two implicit textual techniques demonstrate ZSL’s adaptation to social media: specific phrasing or terminology and genre intertextuality. Specific phrasing introduces extra-media lexical resources, such as the martial term “战胜(*defeated*)” (example 3, line 4) to evoke visceral response, the internet-derived “赞(*Like*)” (example 3, line 14). Genre intertextuality fundamentally reshapes media discourse through conversational lexicon. This includes informal address forms (“we,” “everybody,” “you” in example 3), and popular expressions like “卖个关子(*to create suspense*)” and “小伙伴(*little friends*)” (example 5). Even syntactic features contribute, as interrogatives (example 5, lines 2–3) replace declarative authority with engaging dialog. Collectively, these techniques reframe the audience relationship in social media contexts.

### 4.4 Visual intertextuality of news commentary short videos

**Figure 1:**
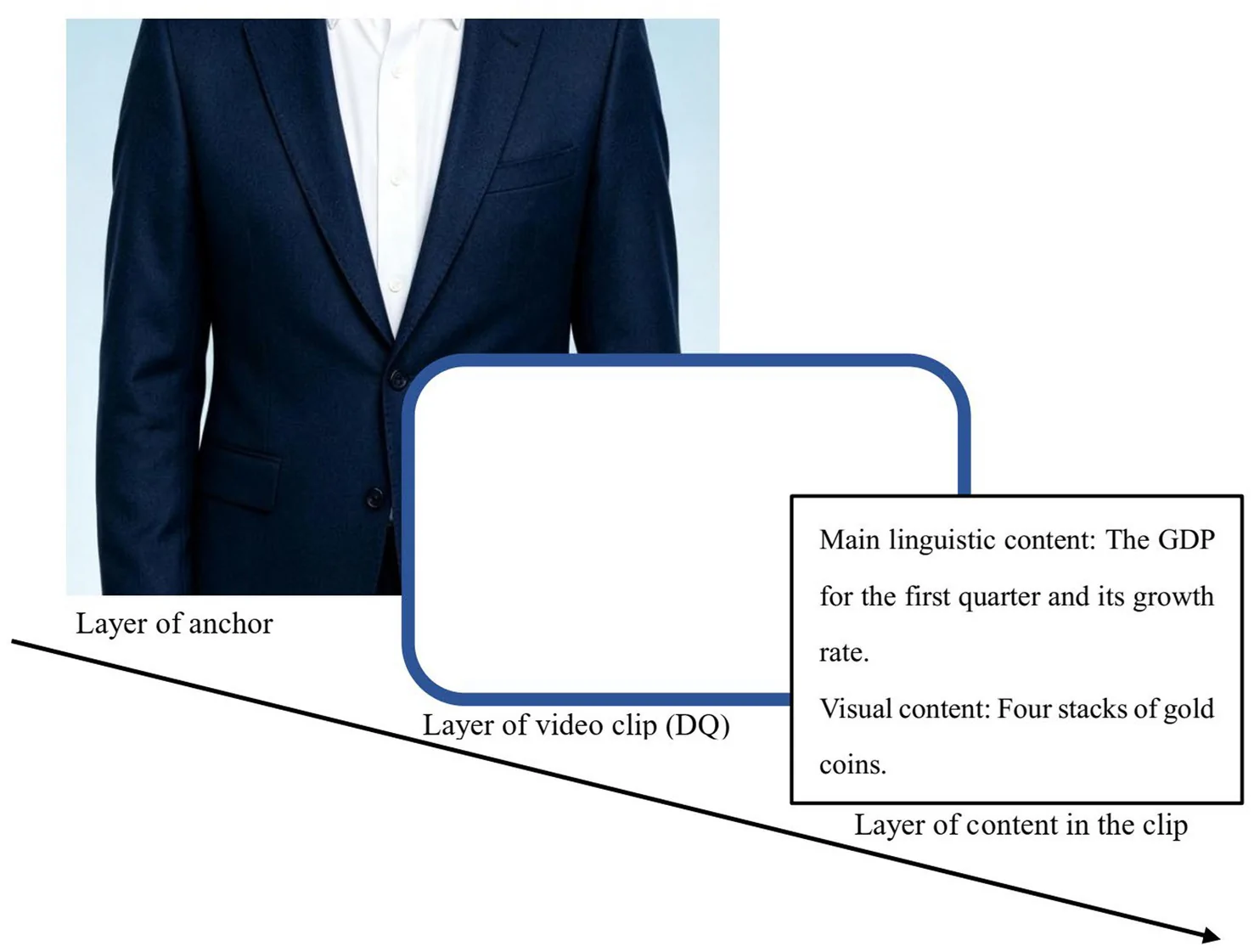
DQ in ZSL (00:04–00:10; Source: 4.5%背后, 是活力满满(*Behind the 4.5% lies abundant vitality*); Date: April 18, 2023).

**Figure 2:**
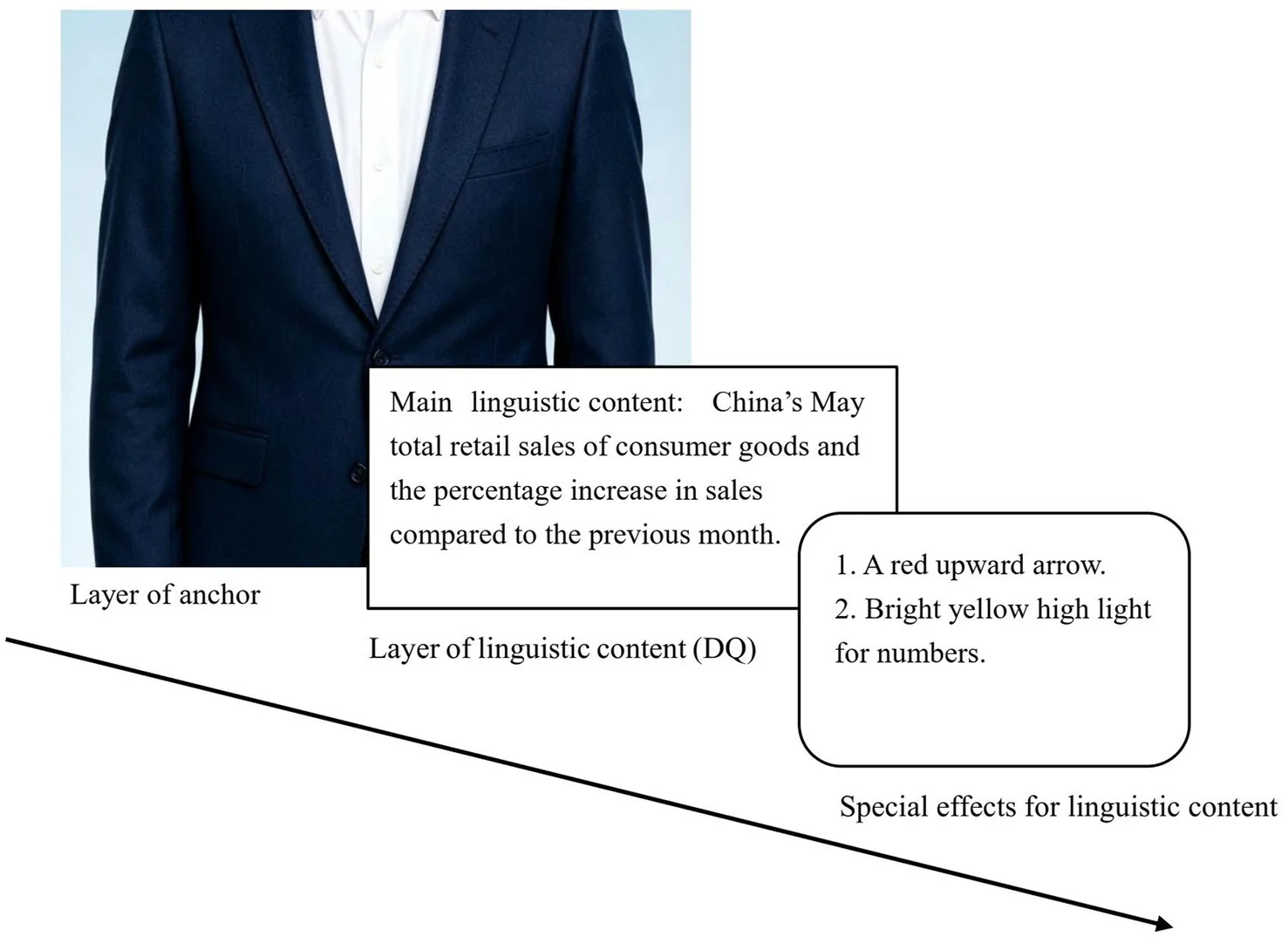
DQ in GCC (00:07–00:10; Source: 国家统计局:5月社会消费品零售总额增长112.7%(*The National Bureau of Statistics: The total retail sales of consumer goods rose by 12.7% in May*); Date: June 23, 2023).

**Figure 3:**
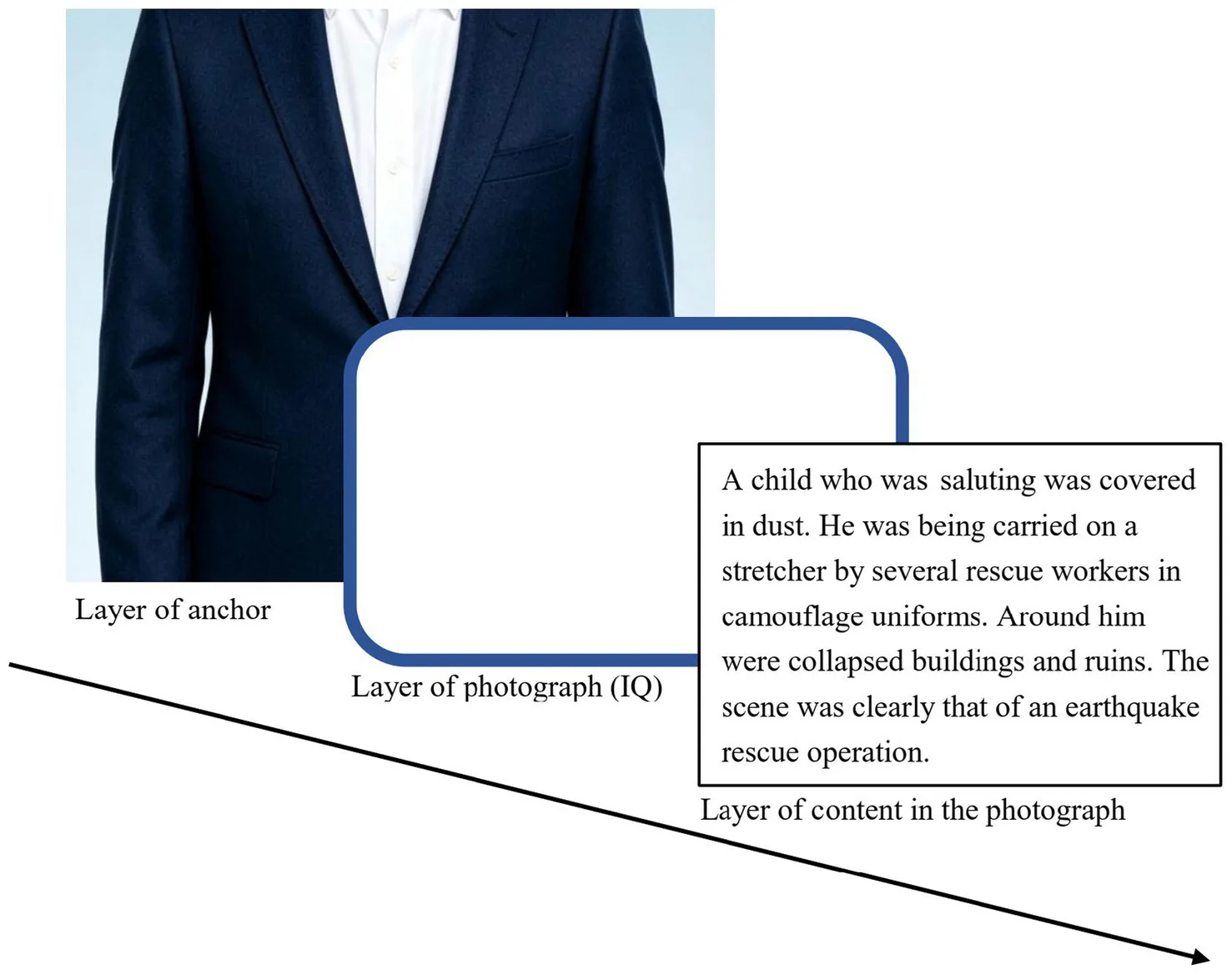
IQ in ZSL (00:08–00:18; Source: “敬礼娃娃”刷屏,这不只是一个温暖的故事(*“The Salute Boy” went viral, and this is not just a touching story*); Date: June 24, 2023).

**Figure 4:**
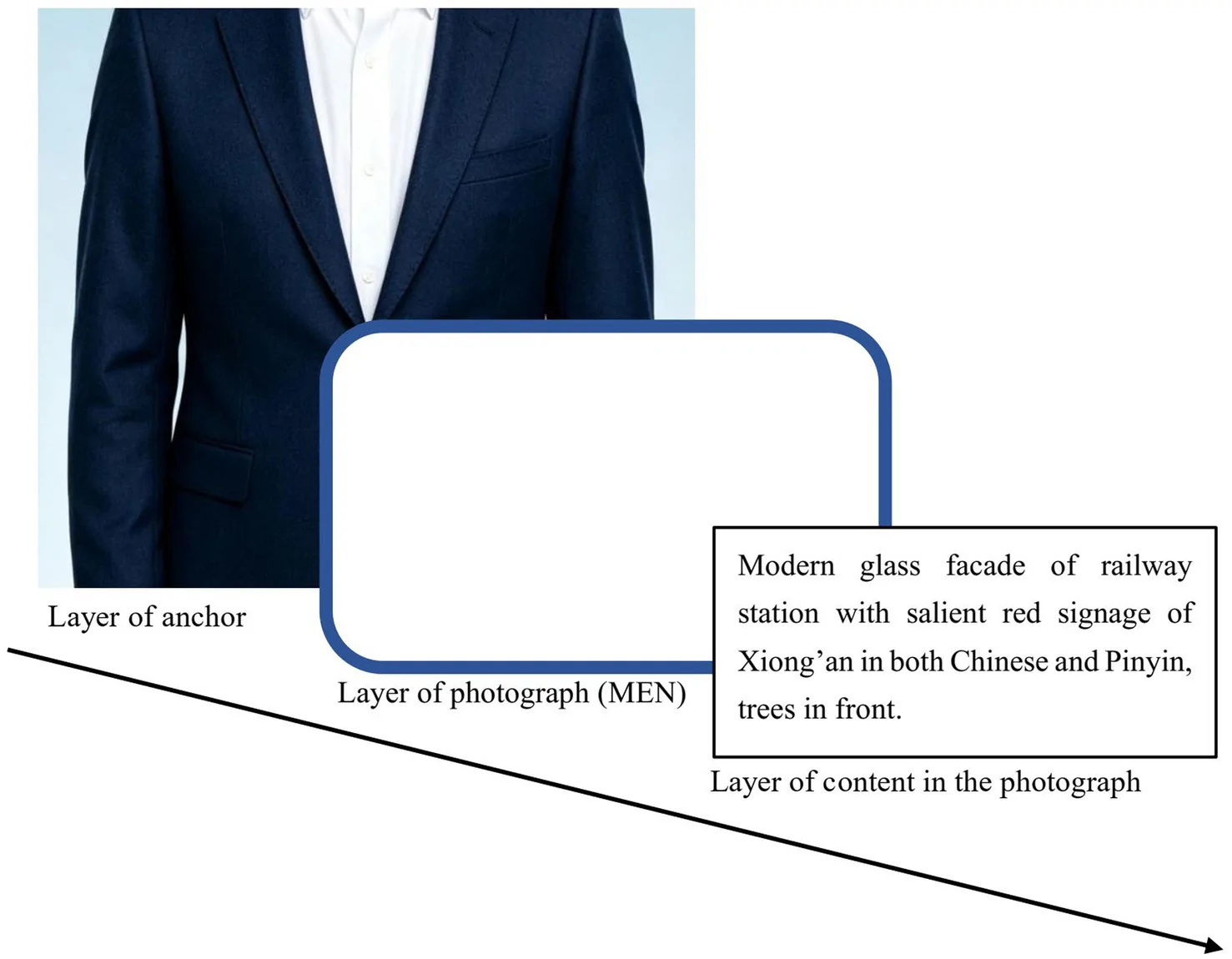
Men in ZSL (00:16–00:31; Source: 中央制定出台一揽子支持政策!未来之城,更加可期(*The central government formulates and introduces a package of supportive policies! The city of the future is even more promising*); Date: June 30, 2023).

**Figure 5:**
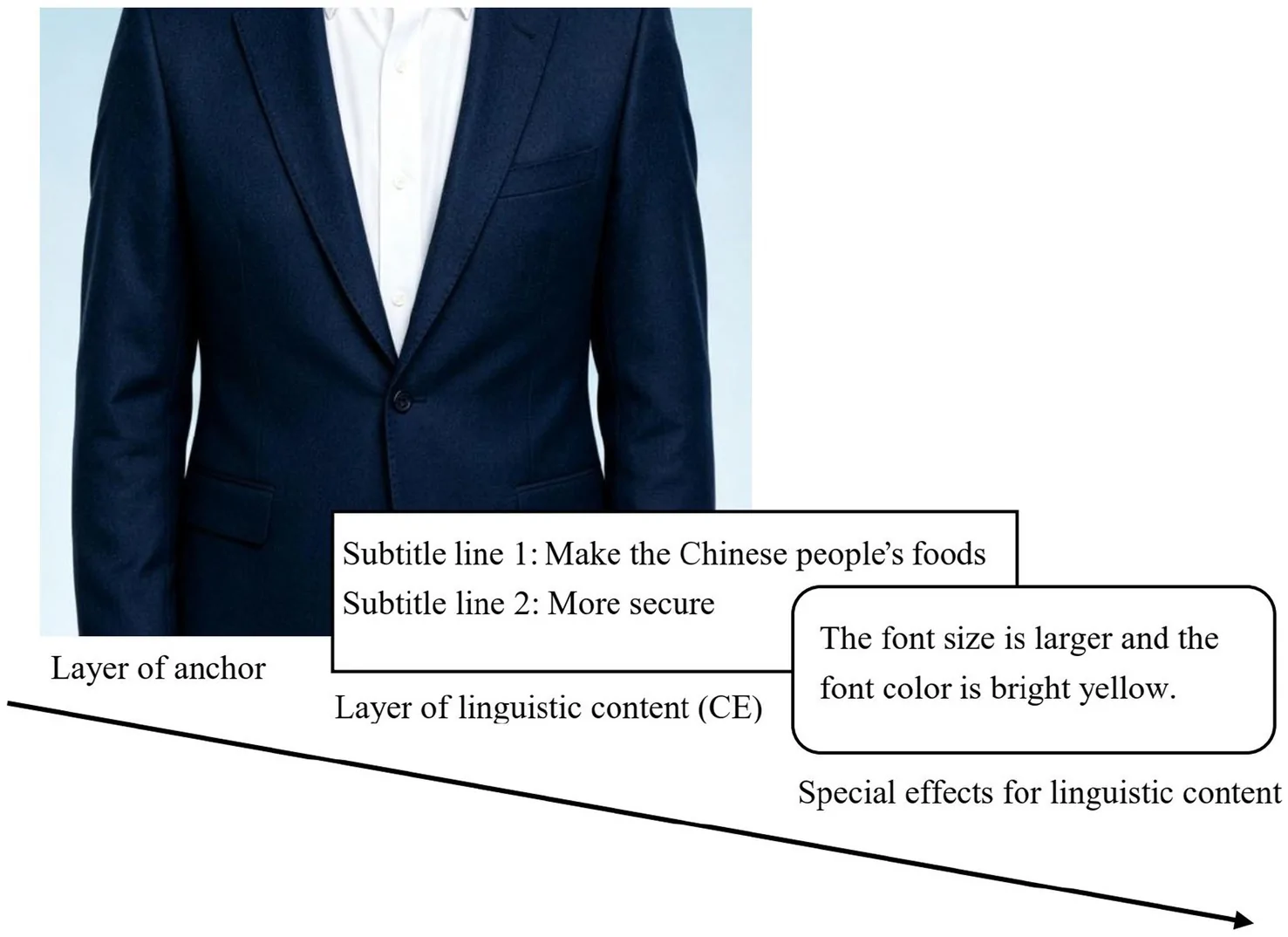
CE in ZSL (02:08–02:10; Source: 这部典籍,值得“典”赞(*This classic work deserves to be highly praised*); Date: February 12, 2023).

**Figure 6:**
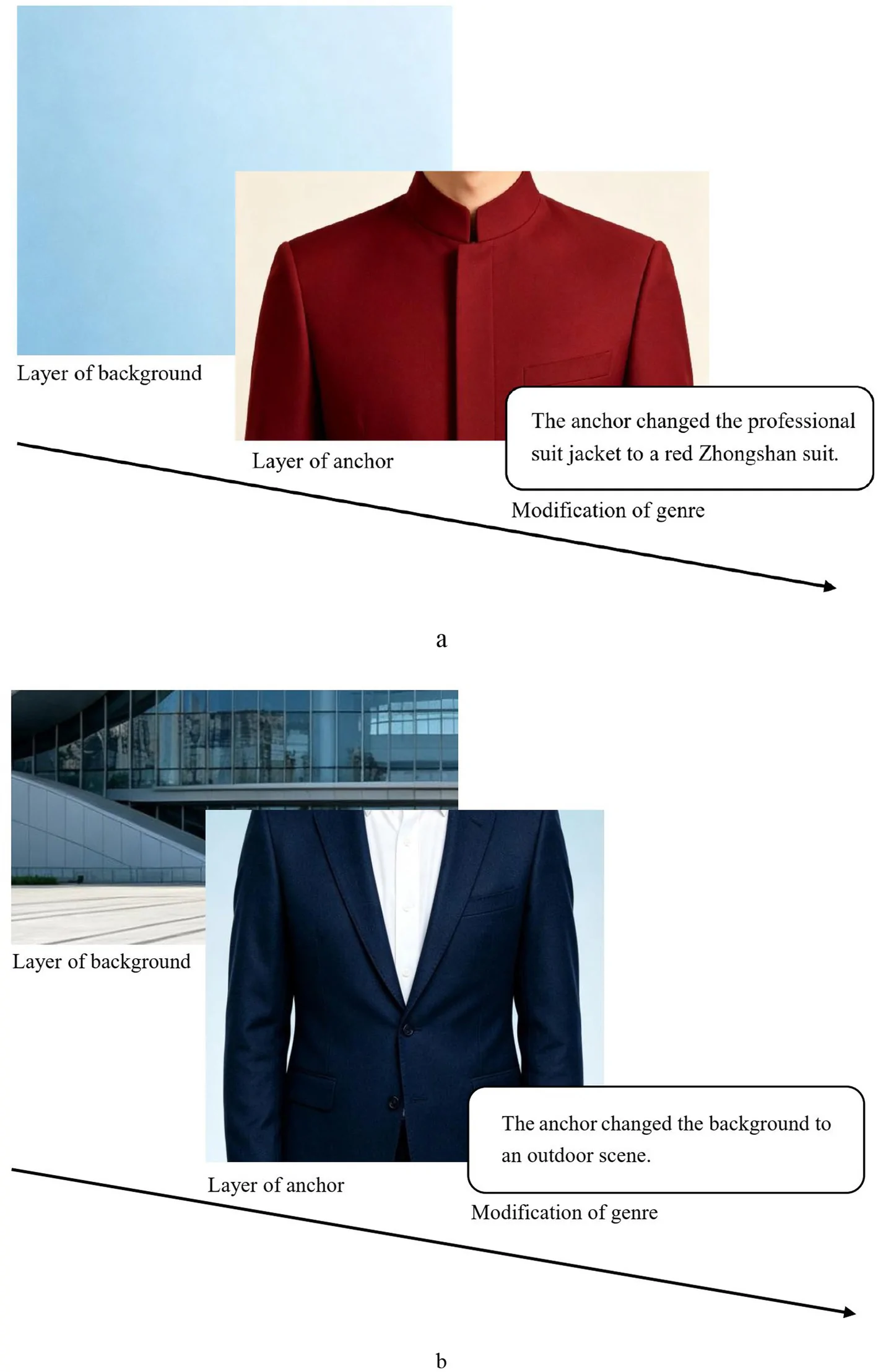
GEN in ZSL (Source **a**: 探月工程,大展宏“兔”!(*The lunar exploration project is about to achieve great success!*); Date: January 26, 2023; Source **b**: 跨越遥远距离,望见美好生活与勃勃生机!(*Across the vast distance, we catch sight of a beautiful life and vigorous vitality!*); Date: April 22, 2023).

Although linguistic intertextuality constitutes the primary mode of source-text recontextualization in both programs, communicative meanings are also shaped through visual resources. According to the overlaid intertextual interaction model proposed by Xing and Feng (2023) in their research, where various multimodal intertextual resources jointly constitute different symbolic practices, this study regards the linguistic mode and the visual mode as the interactive forms of overlapping intertextuality of different modalities. Since the content of news commentary is mainly realized through linguistic mode, and visual mode gradually emerges along with the development of language narrative, there is a close modal interaction between verbal mode and visual mode. The intertextuality of visual symbol resources can provide a more comprehensive explanation for the communicative roles of different presenting entities in the new media context. This section will first present the identified visual mode intertextual techniques, and then further explain how these symbolic resources are further interpreted by the visual grammar analysis framework. Pictures, video clips, and special effects function as main visual intertextual channels through which source symbolic materials are selected, highlighted, and transformed, thereby contributing to the performance of different communicative roles. A fundamental contrast exists between ZSLeco and GCC in their visual intertextual practices. While ZSLeco draws on a relatively broad range of visual resources, including pictures, videos, and subtitle special effects, GCC concentrates primarily on special-effects-based intertextuality, with pictures and video footage appearing only sporadically. The six examples presented in Figures 1–6 illustrate how ZSL and GCC employ visual resources to reproduce, highlight, and transform source materials in different ways including images, video clips and enhancement of special effects in a layered form, in which the colored frame represents the placement of images or video clips, the rectangle indicates the content of the multimodal intertextuality, and the rounded rectangle explains the modification or special effect implemented.

Figures 1, 2 demonstrate the multimodal realization of direct quotation in the two programs. In Figure 1, ZSL incorporates a video clip from the XL into the commentary, using the material as a direct reference to economic data discussed in the source news. In contrast, Figure 2 shows GCC’s use of linguistic special effects to foreground quoted economic information. The upward arrows and highlighted fonts visually emphasize the quoted data. These practices indicate that both programs employ visual resources to reinforce direct quotation, to visually enhance the authority and salience of institutional information.

In Figure 3, when the human anchor discusses the story of “Salute Boy,” a photograph of the individual is displayed. The image itself cannot convey the full event narrative; rather, the meaning of the image is constructed through the accompanying linguistic explanation. Thus, the picture functions as a visual reference that anchors the mentioned individual within the news narrative. As Figure 4 shows, a photograph of Xiong’an Railway Station was introduced in the commentary when the anchor discussed preferential policies toward economic development zones. Although the image does not directly represent the policy content being discussed, the visible geographical marker “Xiong’an (雄安)” establishes a connection between the visual resource and the verbally mentioned content.

Figures 5, 6 illustrate how visual symbolic resources participate in evaluation and genre intertextuality. Figure 5 shows ZSL’s use of subtitle special effects to visually amplify the anchor’s positive evaluation of a news event. The larger and bolder fonts, together with warm-colored visual effects, transform the verbal evaluation into a more salient visual signal, thereby strengthening the expressive and attitudinal dimension of the commentary. Figure 6 demonstrates genre-based intertextuality through the change of conventional news presentation formats. Rather than maintaining the fixed studio-based setting traditionally associated with mainstream television news, ZSL modifies the anchor’s clothing and presentation background according to specific themes. For example, in Figure 6a, the anchor wears a red traditional-style outfit in a New Year-themed program, while in Figure 6b the episode incorporates an on-site background related to the reported topic. These practices extend beyond the transmission of information by reshaping the conventional genre expectations of news commentary, creating a more accessible and audience-oriented mode of presentation.

According to the representative meaning, the six visual examples above show that both human and AI anchors serve as active participants in news commentary videos. In the majority of samples, images of anchors appear with a consistent conceptual representation, dressed formally and meticulously. As the commentary progresses, various intertextual techniques are employed to incorporate images, videos, or special effects that support the textual content of the news discussion. Only a few cases, such as those shown in Figures 6a,b, deviate from this pattern: human anchors change their broadcasting setting or attire, disrupting the stable conceptual representation and attempting to establish an informal dominant role through casual dress. Overall, most samples indicate that mainstream media’s news commentary videos, whether hosted by humans or AI avatars, consistently construct a formal and authoritative image.

In news commentary short videos, the interactive significance of multimodal intertextuality helps clarify the relationship between participants and audiences, further revealing how different commentaries construct distinct communicative roles. Whether human anchors or AI-generated anchor, both directly face the audience throughout the commentary, maintaining continuous eye contact. In contrast, individuals appearing in images or video clips introduced via direct or indirect intertextual techniques are not subject to this direct gaze constraint, such as the boy in Figure 3 who made no direct eye contact with audience. As a result, two types of visual representations emerge: “demand” pictures and “offer” pictures, as defined by Kress and van Leeuwen (2006, p. 117). These two multimodal intertextual resources form an interactive pattern dominated by the anchor, supplemented by image-based intertextual elements. Regarding social distance, human anchors in ZSL appear in close-up half-body shots, while the AI anchor in GCC are shown in long-distance full-body shots, creating differences in perceived intimacy with the audience. The ZSL program thus constructs a more approachable image, whereas GCC appears more distant. From the perspective of attitude conveyed through visual imagery, both programs, including cross-topic samples from ZSL, position the anchors centrally in both horizontal and vertical frames. This central placement highlights the informational importance of the primary visual participant while establishing an egalitarian interactional dynamic with viewers. Furthermore, the centered viewpoint fosters a stable attitudinal resource, aiming to construct a communication role associated with reliable information sources.

In applying the metafunctions of visual grammar to the multimodal intertextuality analysis of news commentary short videos, compositional meaning offers the most comprehensive explanation for the interactive construction of multimodal significance in these videos. It adequately accounts for the informational value, salience, and specific spatial arrangement of multimodal symbolic resources, such as the visual images of anchors from two news commentary programs and the various intertextual techniques employed. Beyond the prominent positioning of anchor images revealed through the first two metafunctions, multimodal intertextual resources in ZSLeco, GCC, and cross-topic ZSL samples are typically placed toward the lower center of the frame, thereby highlighting their informational value and salience without compromising the visual contact with the anchor. Both ZSL and GCC programs enhance the informational value and prominence of symbolic resources such as images and videos by adding borders around them as shown in above examples, regardless of the type of intertextual technique used. This not only distinguishes these symbolic elements from the background formed by the host’s image but also signals that they originate from external sources and belong to the news commentary context. Consequently, both the anchor’s visual image and symbolic resources, including pictures, videos, and enhanced effects, are represented in ZSL and GCC videos, collectively forming the overall narrative structure of the news commentary through conceptual representation analysis.

When the frequency statistics of multimodal intertextual techniques in two news commentary short-video programs are combined with the analysis of visual intertextual meta-functions, the differences in the constructed communicative meanings of the two programs can be more clearly illustrated. Besides the subtle differences in the represented meanings and interactive meanings, the analysis of compositional meaning benefits more from the statistical results, because the images of anchors are relatively stable, while other visual configurations constantly change as the narrative progress of the news commentary unfolds. Therefore, the extensive use of special effects in GCC for the subtitles directly quoting the content is a direct reinforcement of the value and salience of the intertextual content. Since the DQ itself in this program context implies the emphasis and adherence to official data and authoritative sources, the further reinforcement of visual symbols actually shapes the role of GCC program as a financial information reporting rather than a dynamic commentary role, to some extent, differing from its original program intention. While ZSLeco and expanded ZSL data consistently show the diversity of intertextual techniques in linguistic and visual modalities. From the perspective of compositional meaning, the diverse visual symbol resources further shape the image of the ZSL program as placing greater emphasis on evaluation and maintaining a greater social distance from the audience. However, in a few ZSL cases, externally introduced images or video resources via intertextuality completely cover the entire screen, maximizing their value and salience. This compositional approach is commonly used when presenting images of the country’s top leaders and commenting on major political events. Such framing aligns with the classification process of conceptual representation within representational meaning, reconstructing the relationship between active and passive roles in news commentary videos by overlaying images of higher-level participants over those of lower-level ones. According to the classification framework of conceptual representation in representational meaning (Feng, 2023), hierarchical relationships among participants in news commentary videos can be manifested through explicit or implicit multimodal interactions. Although human news presenters in the linguistic mode remain the active agents in ZSL’s narrative, their obscured images suggest that the multimodal intertextual resources in ZSL programs may shift from conceptual representation to narrative representation. However, this modality interaction pattern was not observed in GCC programs, possibly because financial news commentaries are less likely than political ones to involve shifts in participant status. These differences indicate that human-anchored and AI-presented commentaries adopt different approaches to organizing and recontextualizing prior texts, providing a basis for further examining how intertextual practices contribute to the construction of communicative roles.

## 5 Discussion

### 5.1 Communicative roles constructed by multimodal intertextuality

Research questions 2 and 3 will be answered in the discussion. The results of the meta-functional analysis based on the above multimodal intertextuality can be seen that, in general, whether it is the news commentary video program featuring human or AI anchors under the financial theme or the news commentary program featuring human hosts across multiple themes, the image of the anchor, as the basic represented participant, shows many common points in terms of representational meaning and interactive significance. That is, the anchor and their language delivery are shaped into a reliable, formal, and authoritative image. In contrast, ZSLeco and GCC have significant multimodal intertextual differences in compositional meaning. Consistent with Mellado (2015) conception of journalistic roles as discursively performed rather than merely institutionally prescribed, the human-anchored and AI-presented programs employ intertextuality not simply as a means of incorporating external sources but as a resource for enacting different relationships among source texts, news presenters, and audiences. While ZSLeco demonstrates diverse balanced multimodal intertextual practices, it not only directly and indirectly quotes authoritative information, but also actively provides comments, reduces the difficulty of understanding through non-professional terms, and further makes the news commentary content closer to the audience by changing the traditional preaching genre style of mainstream media. This, to a certain extent, balances authority and closeness, and pays attention to the understanding of non-professional viewers of news in a more equal communicative role. This result still holds after expanding the ZSL sample library. In different news commentary themes, ZSL still demonstrates diverse multimodal intertextual techniques, by actively providing evaluative content to re-contextualize the intertextual source information.

A defining characteristic of ZSL is its extensive use of transformative intertextuality. Rather than functioning as a neutral conduit for pre-existing information, the human anchor actively selects, reorganizes, and interprets source materials through indirect quotation, mentioning, comment and evaluation, genre intertextuality, and multimodal integration. This role is evident in the way institutional voices are incorporated into commentary. Authority strategies systematically employ direct quotation to “graft” institutional credibility from traditional media sources into new media contexts, aligning with van Leeuwen (2007) concept of legitimization through authority and what Fairclough (1992, p. 119) identifies as “using the authority of an external voice to bolster one’s position.” Direct quotation therefore does not merely reproduce external voices; it functions as a mechanism through which institutional authority is recontextualized and made meaningful within a new communicative setting. At the same time, ZSL frequently moves beyond simple reproduction of source materials through positioning strategies. Commenting, evaluating, indirect quotation, and mentioning allow the anchor to actively construct and reinforce particular stances toward reported events. In this sense, the anchor performs what Bazerman (2004, p. 86) describes as “using the voices of the people and groups they report on to tell their own stories, just as novelists use characters or ventriloquists use dummies.” The source text is not simply relayed but transformed into a new discourse that organizes, prioritizes, and interprets information for audiences. Through this process of recontextualization, the anchor assumes the role of a meaning-maker who bridges institutional discourse and public understanding.

Genre intertextuality further strengthens this mediating role by connecting official discourse to broader cultural and everyday communicative practices. Rather than maintaining the formal style associated with traditional news broadcasting, ZSL frequently incorporates colloquial expressions, popular cultural references, and visually adapted presentation formats. Genre intertextuality thus allows institutional discourse to be reorganized into more accessible communicative forms. In this respect, the findings resonate with Gong et al. (2023) observation that contemporary mainstream media increasingly employ “softened” communication that “portrays ordinary people and other social roles as well.” Through informal address forms, conversational language, and thematic visual settings, ZSL reduces the symbolic distance between institutional media and audiences, reshaping the conventional one-way transmission model into a more dialogic communicative relationship. Taken together, these intertextual practices construct a communicative role centered on interpretation, contextualization, and audience engagement.

According to the comparison results presented in Table 2 and Table 4, the financial news commentary presented by AI almost exclusively uses direct citation of language modality to visually configure the multimodal intertextual content, and the enhanced special effects in the picture are mainly based on the adjustment of the text form. These intertextual resources further strengthen the authoritative image of the AI-presented host, and construct the AI-presented news commentary video as a simple information transmitter role. AI-presented GCC exhibits a markedly different intertextual profile that gives rise to a different communicative role. Whereas ZSL is characterized by transformation and reinterpretation, GCC relies predominantly on the reproduction of source materials through direct quotation and the visual highlighting of quoted information. The relative absence of evaluative commentary, indirect reformulation, and genre-based adaptation suggests a communication mode oriented toward source fidelity and institutional consistency rather than interpretive intervention. GCC’s quotation-centered intertextuality reflects a communicative logic in which authority is derived primarily from the accurate transmission of existing institutional discourse. Rather than positioning the presenter as an interpreter of events, this approach foregrounds the source itself and minimizes the visibility of the presenter’s own voice. This role can be understood as that of an institutional information relay. The AI presenter functions less as a mediator who transforms discourse and more as a channel through which authoritative information is circulated across media platforms. Such a role is consistent with the characteristics of AI-generated communication identified by Tang (2025), who describes AI-generated text as a “mosaic of quotations” reconfigured from training data. Although the content analyzed in this study was reviewed and published within a mainstream media workflow, GCC nevertheless demonstrates how AI-presented commentary tends to organize texts. The result is a communicative role that emphasizes consistency, standardization, and institutional alignment rather than extensive contextualization or interpretive elaboration.

Viewed together, the analysis of multimodal intertextuality also shows the synergy between the language modality and visual modality of news commentary, that is, the language content of the news commentary as the main narrative mode, on the one hand, establishes the active participant identity of the human or AI image, and on the other hand, the visual symbol resources have a reinforcing effect on the construction of the communicative role. This modality synergy conforms to the viewpoints of Kress and van Leeuwen (2006) and Jewitt (2009). Moreover, these findings demonstrate that communicative roles in contemporary news commentary are not predetermined by the identity of the presenter alone but are actively constructed through specific patterns of intertextual practice.

### 5.2 Human-AI division of communicative labor in news commentary

The contrasting communicative roles identified above suggest that the relationship between human and AI presenters is better understood as complementary rather than competitive. Rather than indicating that one mode of communication is inherently superior to the other, the findings point to different strengths emerging from different intertextual configurations. Human anchors primarily contribute through interpretation, evaluation, contextualization, and audience engagement. By selectively incorporating quotations, reformulating source materials, introducing evaluative perspectives, and drawing upon broader cultural resources, they transform information into socially meaningful narratives. Such practices enable human presenters to perform what Mellado (2015) conceptualizes as role performance, actively shaping how news events are understood rather than merely transmitting information. In contrast, AI presenters contribute through information reproduction, rapid dissemination, standardized delivery, and institutional consistency. GCC’s quotation-centered intertextuality demonstrates a communicative orientation toward preserving source fidelity and ensuring the stable circulation of authoritative information across platforms. While AI anchors undeniably offer compelling cost efficiencies, requiring primarily “technical maintenance and software updates, without high salary and training costs” (Li, 2025, p. 6), their value in news commentary lies less in interpretive intervention than in the efficient processing and presentation of information.

This distinction helps explain why human and AI presenters currently occupy different positions within contemporary news ecosystems. The findings suggest that the strengths of AI communication are concentrated in tasks involving large-scale information processing, retrieval, and standardized presentation, whereas human communicators retain particular importance in activities requiring contextual judgment, evaluative framing, and the transformation of institutional discourse into audience-oriented communication. Existing research has noted a number of challenges facing AI presenters, including limitations in emotional expression, audience concerns regarding authenticity, and the risk of information overload (Li, 2025, p. 8–11). Rather than interpreting these issues simply as technological shortcomings, the present findings suggest that they reflect differences in communicative specialization. While AI anchors excel at efficiently relaying information, achieving genuine user-specific interactivity and contextual intelligence necessitates much deeper collaboration between human and AI communicators informed by systematic understanding of how different intertextual resources function in news discourse.

From this perspective, the increasing integration of AI into journalism does not imply the disappearance of journalistic labor but rather its redistribution. Traditional journalism often concentrated information gathering, interpretation, evaluation, and presentation within a single professional role. The emergence of AI communicators increasingly separates these functions. Human journalists are likely to become more involved in meaning construction, value framing, contextual explanation, and communicative adaptation, while AI systems assume a growing role in information processing, retrieval, organization, and standardized presentation. This emerging division of labor aligns with broader arguments that AI should be understood not merely as a technological tool but as a communicative actor within news production processes (Lewis et al., 2019; Guzman and Lewis, 2020). The coexistence of ZSL and GCC within the same institutional environment illustrates how these roles may operate in parallel rather than in opposition.

Importantly, the findings do not suggest that future news production should move toward either fully human-centered or fully AI-centered communication. Instead, the most promising developmental trajectory appears to leverage AI’s demonstrated efficiency in authoritative information processing and retrieval while strategically reserving more sophisticated transformative intertextual work for human expertise and judgment. Such an arrangement would allow mainstream media organizations to maintain what Ren et al. (2023, p. 6) term the “professionalism and authority of media in explaining problems” while simultaneously adapting communication to the expectations of increasingly fragmented and interactive digital audiences. As AI systems continue to develop toward what Li (2025) describes as the ability to analyze user data and provide increasingly customized news content, the challenge will be not simply improving technological performance but determining how human and AI communicators can most effectively collaborate. The findings of this study suggest that this collaboration is likely to be most productive when AI assumes responsibilities associated with information management and dissemination, while human communicators remain responsible for the interpretive and contextual work through which news acquires broader social meaning.

## 6 Conclusion

The findings reveal two distinct intertextual profiles. While ZSL adopts a diverse and transformative repertoire involving quotation, evaluation, genre resources, and multimodal integration, GCC relies primarily on the reproduction and visual highlighting of authoritative information. These contrasting intertextual configurations construct different communicative roles, positioning the human anchor as an interpretive mediator and the AI presenter as an institutional information relay. Theoretically, the study adapts the multimodal intertextuality analysis to AI-mediated news discourse and demonstrates that communicative roles are actively constructed through multimodal intertextual practices rather than determined solely by presenter identity. The findings also carry practical implications for human-AI collaboration in journalism. Rather than functioning as competing communicators, human and AI presenters appear to perform complementary communicative functions. The results therefore suggest that future news production may benefit from a collaborative division of communicative labor that combines the strengths of both human and AI communicators. This study is limited by its focus on two programs within the same media system, a restricted time period and topic range, which may limit the generalizability of the findings. Future research could extend the comparison to a wider range of media organizations and investigate how audiences perceive and respond to different intertextual configurations in increasingly hybrid human-AI news environments.

## Data Availability

The raw data supporting the conclusions of this article will be made available by the authors, without undue reservation.

## Footnotes

1
https://www.yangshipin.cn/

2
https://tv.cctv.com/lm/xwlb/

## References

[ref1] Ai C. Chen B. He L. Lai K. Qiu X. (2018). The National Geographic characteristics of online public opinion propagation in China based on WeChat network. GeoInformatica 22, 311–334. doi: 10.1007/s10707-017-0311-4

[ref2] Bakhtin Mikhail. (1986). Speech Genres and Other Late Essays. Edited by Emerson C. Holquist M. Translated by McGee V. W. Austin, TX: University of Texas Press.

[ref3] Bazerman C. (2004). “Intertextuality: how texts rely on other texts,” in What Writing Does and How It Does It: An Introduction to Analyzing Texts and Textual Practices, eds. Bazerman C. Prior P. (Mahwah, NJ: Lawrence Erlbaum Associates), 83–96.

[ref4] Bhatia V. K. (2010). Interdiscursivity in professional communication. Discourse Commun. 4, 32–50. doi: 10.1177/175048130935120

[ref5] Bhatia A. (2018). Interdiscursive performance in digital professions: the case of YouTube tutorials. J. Pragmat. 124, 106–120. doi: 10.1016/j.pragma.2017.11.001

[ref6] Bremner S. (2008). Intertextuality and business communication textbooks: why students need more textual support. Engl. Specif. Purp. 27, 306–321. doi: 10.1016/j.esp.2008.01.001

[ref7] Charteris-Black J. (2004). Corpus Approaches to Critical Metaphor Analysis. Houndmills, UK: Palgrave Macmillan.

[ref9] Fairclough N. (1992). Discourse and Social Change. Cambridge, UK: Polity Press.

[ref10] Feng D. (2023). Multimodal Chinese Discourse: Understanding Communication and Society in Contemporary China. London: Routledge.

[ref11] Forstall C. W. Scheirer W. J. (2019). Quantitative Intertextuality: Analyzing the Markers of Information Reuse. Cham, Switzerland: Springer.

[ref12] Fowler R. (1991). Language in the News: Discourse and Ideology in the Press. London: Routledge.

[ref13] Global Times (2022) “China Media Group has joined hands with DeepBrain AI to create the first ultra-realistic AI anchor.” Available online at: https://finance.huanqiu.com/article/47Kswplzxlr (Accessed July 1, 2024)

[ref14] Gong J. Firdaus A. Aksar I. A. Alivi M. A. Xu J. (2023). Intertextuality and ideology: social actor’s representation in handling of COVID-19 from China daily. Journalism 24, 2741–2761. doi: 10.1177/14648849231157243

[ref15] Guzman A. Lewis S. (2020). Artificial intelligence and communication: a human-machine communication research agenda. New Media Soc. 22, 70–86. doi: 10.1177/1461444819858691

[ref9002] Halliday M. A. K. (1994). An introduction to functional grammar. 2nd Edn. London: Edward Arnold.

[ref16] Hart C. (2017). Metaphor and intertextuality in media framings of the (1984-1985) British miners’ strike: a multimodal analysis. Discourse Commun. 11, 3–30. doi: 10.1177/1750481316683291

[ref17] Hou J. (2021). Our service’: normalizing CCTV’S institutionalized practices through Chunyun discourse. Journalism 22, 1851–1869. doi: 10.1177/1464884919828508

[ref18] Jewitt C. (2009). “An introduction to multimodality,” in The Routledge Handbook of Multimodal Analysis, ed. Jewitt C. (London: Routledge), 14–27.

[ref19] Kazmierczak M. (2019). Intertextuality as translation problem: explicitness, recognisability and the case of ‘literatures of smaller nations’. Russ. J. Linguist. 23, 362–382. doi: 10.22363/2312-9182-2019-23-2-362-382

[ref20] Kim N.-y. (2023). A study on the intertextuality of Russian media: focusing on the analysis criteria for intertextuality. Pragmatics Soc. 14, 869–882. doi: 10.1075/ps.22055.kim

[ref21] Krennmayr T. (2014). What Corpus linguistics can tell us about metaphor use in newspaper texts. Journal. Stud. 16, 530–546. doi: 10.1080/1461670X.2014.937155, 37339054

[ref22] Kress G. (2010). Multimodality: A Social Semiotic Approach to Contemporary Communication. London: Routledge.

[ref23] Kress G. van Leeuwen T. (2006). Reading Images: The Grammar of Visual Design. 2nd Edn London: Routledge.

[ref24] Kristeva J. (1969). Recherches pour une Sémanalyse. Paris: Seuil.

[ref25] Kristeva J. (1986). “Word, dialogue and novel”, in The Kristeva Reader, J. Kristeva, ed. T. Moi (New York: Clumbia University Press), 34–61.

[ref26] Lausberg H. Sloetjes H. (2009). Coding gestural behavior with the NEUROGES-ELAN system. Behav. Res. Methods Instrum. Comput. 41, 841–849. doi: 10.3758/BRM.41.3.841, 19587200

[ref27] Lewis S. Guzman A. Schmidt T. (2019). Automation, journalism, and human-machine communication: rethinking roles and relationships of humans and machines in news. Digit. J. 7, 409–427. doi: 10.1080/21670811.2019.1577147

[ref28] Li N. (2024). Analyzing the complexity of public opinion evolution on Weibo: a super network model. J. Knowl. Econ. 16, 3404–3439. doi: 10.1007/s13132-024-02059-9

[ref29] Li L. (2025). Impact of AI virtual anchors on traditional news anchors. Int. J. Knowl. Manag. 21, 1–17. doi: 10.4018/ijkm.362624

[ref30] Liu Y. Yao S. Xi T. Lu B. Zheng W. (2025) “Human vs AI: How Digital Human News Anchors Affect Our Cognitive Processes?” *Proceedings of the 33rd ACM International Conference on Multimedia* 1–10 New York ACM

[ref31] Mellado C. (2015). Professional roles in news content: six dimensions of journalistic role performance. J. Theol. Stud. 16, 596–614. doi: 10.1080/1461670X.2014.922276

[ref32] Mellado C. van Dalen A. (2014). Between rhetoric and practice. Explaining the gap between role conception and performance in journalism. J. Theol. Stud. 15, 859–878. doi: 10.1080/1461670X.2013.838046

[ref33] Morgan C. A. (2019). Intertextuality, Manipulation and Propaganda: Reworking the Arthurian Legend in Contemporary Spanish Literature.” PhD diss. Cardiff University.

[ref34] Ren S. Gong C. Zhang C. Li C. (2023). Public opinion communication mechanism of public health emergencies in Weibo: take the COVID-19 epidemic as an example. Front. Public Health 11:1276083. doi: 10.3389/fpubh.2023.1276083, 38026415 PMC10665477

[ref35] Saraireh M. A. Saraireh T. M. (2020). Intertextuality manipulation in post-September-eleven American fiction to misinform readership. Indones. J. Islam Muslim Soc. 10, 271–296. doi: 10.18326/ijims.v10i2.271-296

[ref36] Tang K. (2025). AI-textuality: expanding intertextuality to theorize human-AI interaction with generative artificial intelligence. Appl. Linguis. 47, 353–371. doi: 10.1093/applin/amaf016

[ref37] van Leeuwen T. (2005). Introducing Social Semiotics. London: Routledge.

[ref38] van Leeuwen T. (2007). Legitimation in discourse and communication. Discourse Commun. 1, 91–112. doi: 10.1177/1750481307071986

[ref39] Velykoroda Y. Moroz O. (2021). Intertextuality in media discourse: a reader’s perspective. Explorations in English Language and Linguistics 9, 56–79. doi: 10.2478/exell-2022-0003

[ref40] Vološinov V. (1986). Marxism and the Philosophy of Language. Translated by Matejka L. Titunik I. R. Cambridge, MA: Harvard University Press.

[ref41] Wells A. R. (2023). Guarding against Extremism in the 21st Century: A Lesson from the Past. German Public Opinion and Hitler’s Policies, 1933–1939. Bloomington: Xlibris Us.

[ref42] Wiederhold B. K. (2019). Animated news anchors: where to next? Cyberpsychol. Behav. Soc. Netw. 22, 675–676. doi: 10.1089/cyber.2019.29167.bkw, 31697603

[ref43] Xing C. Feng D. (2023). Multimodal intertextuality and persuasion in advertising discourse. Discourse Commun. 17, 613–629. doi: 10.1177/17504813231170579

[ref44] Xinhua News Agency (2015) “Opinion on Strengthening the Building of New-Type Think Tanks with Chinese Characteristics.” Available online at: http://www.gov.cn/xinwen/2015-01/20/content_2807126.htm (Accessed January 20, 2015)

[ref45] Xue K. Li Y. Jin H. (2022). What do you think of AI? Research on the influence of AI news anchor image on watching intention. Behav. Sci. 12:465. doi: 10.3390/bs12110465, 36421761 PMC9687615

